# Using a units ontology to annotate pre-existing metadata

**DOI:** 10.1038/s41597-025-04587-8

**Published:** 2025-02-20

**Authors:** John H. Porter, Margaret O’Brien, Marina Frants, Stevan Earl, Mary Martin, Christine M. Laney

**Affiliations:** 1 University of Virginia, Charlottesville, Virginia USA; 2 University of California, Santa Barbara, Santa Barbara, California USA; 3 Scripps Institution of Oceanography, La Jolla, California USA; 4 Arizona State University, Tempe, Arizona USA; 5 University of New Hampshire, Durham, New Hampshire USA; 6 National Ecological Observatory Network (NEON), Battelle, Boulder, Colorado USA

**Keywords:** Ecological networks, Databases

## Abstract

Automated processing of environmental data is hindered by the wide array of unit representations provided in the metadata of digital datasets. For example, gm/m2, g/m2, gm-2, g/m^2, g.m-2 and gramPerMeterSquared are all representations of a single complex unit that might be human-readable but are not machine-interpretable. Connecting *ad hoc* units to a single unit concept in an ontology permits the identification of datasets sharing units and provides additional information regarding labels, definitions, dimensions and transformations provided in the ontology. Here we use successive string transformations to link *ad hoc* unit representations to units in the QUDT ontology (e.g., unit: GM-PER-M2). Although only 896 of 7,110 distinct units in a corpus of ecological metadata from DataONE, the Environmental Data Initiative and the U.S. National Ecological Observatory Network were matched, 324,811 unit uses (instances) out of 355,057 of total unit uses were successfully mapped to QUDT units (91%). The resulting lookup table was used to enable a web service and R functions for adding annotation elements to Ecological Metadata Language documents.

## Introduction

Numbers without units are not data, they are just numbers. This was never more apparent than when the Mars Climate Orbiter burned in the Martian atmosphere due to one planning group working in metric units and another in English units^1^, or when an aircraft ran out of fuel half-way through a scheduled flight and glided to a landing because fuel had been loaded in pounds instead of kilograms^2^. Units form the basis for the Unit Factor Method or Dimensional Analysis, which is an important scientific tool for assessing the plausibility of derived equations and for allowing conversions between systems of units^3^. Although the importance of units is well established in science, their expression is often irregular and *ad hoc*, especially in datasets of primary research results. Here we present results from an effort to enhance a large corpus of existing environmental metadata through linking existing unit representations with standard unit identifiers coupled to an ontology.

Hanisch *et al*.^4^ made a call to “Stop squandering data: make units of measurement machine-readable.” They reviewed some of the challenges associated with current use of units including situationally dependent unit representations (where a given unit notation has different meanings in different contexts), the use of different unit representations to express the same quantity, and overloading where the same letter can be used to represent multiple, different units. These issues are exacerbated for automatically processed digital data, where human judgment and experience are lacking. As they noted: “Unless we take steps to ensure that measurement units are routinely documented for easy, unambiguous exchange of data, information will be unusable or, worse, be misinterpreted.”

Although issues with units and unit conversions are widespread, ecological and environmental data are particularly fraught due to the diversity of units needed to address physical drivers (e.g., climate, weather, soil characteristics), a wide variety of organisms and their characteristics (e.g., microbes, plants, insects, vertebrates), and chemical processes (e.g., carbon and nitrogen cycles) across a similarly wide array of environments and scales (e.g., polar, oceanic, desert, forest, grassland, coastal, aquatic, atmospheric). Moreover, disparate unit representations for identical units are common. For example, in the area of microbial research, the unit “per mil” is widely used whereas “parts per thousand” might be more acceptable elsewhere, but both are equivalent, dimensionless units for a concentration. With the need to link primary environmental research results with promising climate models, it is increasingly desirable that there be consistent machine-readable units applied to data both within and across datasets and research networks.

A truism attributed to legendary computer pioneer Grace M. Hopper is that “The wonderful thing about standards is that there are so many of them to choose from”^5^. This definitely applies to units. There are many systems of units (e.g., metric, English, Imperial) and within those are many different ways of representing units. A challenge for users is that unit representations often contain very few characters (e.g., mg could mean milligram, magnesium, milligravities, or, if capitalized, a brand of car, the disease Myasthenia gravis, or megagauss), and the unit system may be discernible only from context. A challenge for data users when confronted with an ambiguous unit representation is to identify which system is being used and then which of the many options for representing a unit is being employed.

Here we attempt to address the challenge posed by diverse units and disparate representations in environmental metadata. We present the process by which uncontrolled, *ad hoc* and often irregular unit descriptions, hereafter described as “raw units”, in existing metadata from three sources – the Environmental Data Initiative (EDI)^6^, the National Ecological Observatory Network (NEON)^7^, and DataONE^8^– were linked to an extant unit ontology, and provide methods for annotating metadata to provide a consistent human and machine-readable representation of units. To control the scope of the current work, our focus here is on the raw unit description given by the metadata provider without vetting a dataset constructor’s unit choice. We do not attempt to determine if a raw unit was the correct one for the measurement it was attached to, but rather to link raw units to machine-readable forms.

There are several frameworks being used to promote data interoperability in the context of units. Early standards-based attempts, such as ISO 2955, ANSI X3.50 and ENV 12435.7, focused primarily on human-readable and printable representations of units^9^. Several more recent efforts, such as the Unified Code for Units of Measure (UCUM)^10^, the Units of Measure (OM) ontology^11^, and the Quantities, Units, Dimensions and Types (QUDT) ontology^12^ focus on machine-readability.

The Unified Code for Units of Measure (UCUM), which provides syntax and lexical rules for describing units, focuses primarily on electronic communication^10^. UCUM provides tables of standardized prefixes for powers of 10 (e.g., G for 10^9^), metric base units for length, time, mass, plane angle, temperature, electric charge and luminous intensity with print, case sensitive and case insensitive versions for each, and lists of common unit codes. These are linked to other unit systems (e.g., customary units, such as inch and yard), again with standard definitions. Examples of UCUM codes are cm for centimeters, kPa for kilopascals, and mg/min for milligrams per minute. UCUM has been adopted by many organizations, especially in the biomedical realm, with several organizations providing unit lists for commonly used units and web services for unit conversion and validation. However, UCUM is rules-based, and does not have an underlying ontology to help link unit representations to additional information.

The OM version 2.0 ontology has over 1,300 units with information on quantities, measures, and dimensions, focused primarily on scientific domains^11^. OM provides labels, comments, and the associated International System of Units (SI) unit and corresponding multiplication factor for conversion to SI.

QUDT integrates Quantity, Unit, Dimension and Type ontologies to provide standard unit representations linked to their underlying properties such as dimensions and multiplier to SI units. QUDT includes a class “QuantityKind”, the observable property linking the measurement to its unit. It also includes alternative representations (such as UCUM codes) and for some QuantityKinds, equivalent URIs for entries in the SI Digital Framework (https://si-digital-framework.org/, accessed 2024-12-19)^13^. Unlike UCUM where new, valid, derived units can be created on-the-fly, QUDT has a formal process for adopting new units based on syntax rules for creating the unit code linked to properties such as labels, multipliers to convert to SI units, quantity/kind (e.g., Density, MassPerArea, LinearVelocity) and the relevant dimensions (i.e., amount of a substance, electric current, length, luminous intensity, mass, thermodynamic temperature, time and dimensionless). Quantity/kind and dimensions each have their own related ontology.

Keil and Schindler^14^ reviewed 8 different unit ontologies, including OM, and an early version of QUDT. They found very little overlap in the units addressed, with only 17 units occurring across all ontologies, and fewer than 75% of units were common among pairs of ontologies. Such differences are expected given the diversity of the projects and scientific domains from which they originated.

Here we chose to use the QUDT ontology because it provided: (1) a more complete coverage of biological and environmental units than most other ontologies; (2) a URI that could be used in a Resource Discovery Framework (RDF) triplet; (3) a relatively large amount of ancillary information about a unit, including labels, text descriptions, a multiplier and optional intercept to aid in unit conversions, information on the dimensional components and their relationships; and (4) identifiers for the same unit in other systems (e.g., Ontology of Units of Measure, IEC61360, UNECE and UCUM). Moreover, QUDT seems to have a vibrant and active community developing new unit descriptions using a logical and thoughtful framework.

## Methods

In our analysis we will refer to several different types of units or groups of units. These are:

- **Raw Units**: These are the original unit descriptions provided by metadata providers. These are uncurated and *ad hoc*. The list of raw units includes many duplicates.
- **Distinct Units**: Raw units with duplicates removed
- **Pseudounits**: An edited form of distinct units (lowercase, no spaces, symbols replaced)
- **QUDT Units**: Units included in the QUDT ontology

**Fig. 1:**
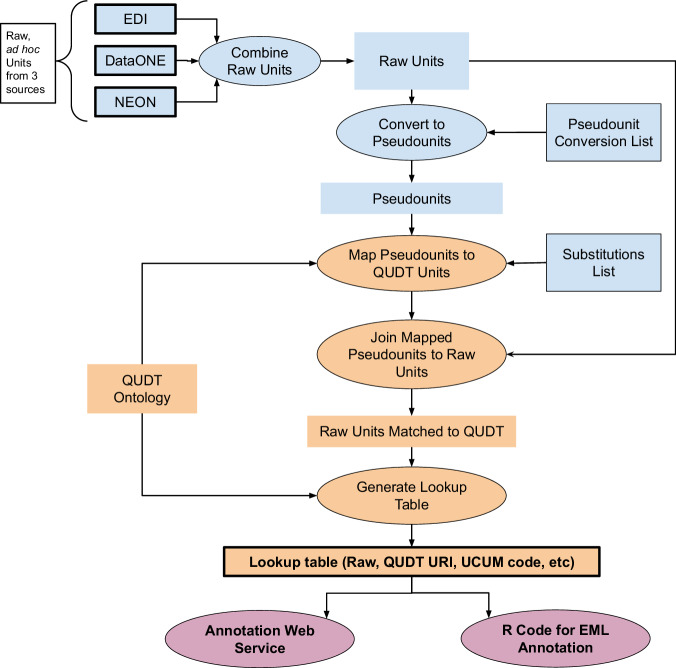
Steps to create and process raw units to a final lookup table that includes QUDT mappings with web services. Colors represent phases: Blue: initial processing to convert raw units to pseudounits. Orange: creation of final lookup table. Magenta: web services for augmenting metadata with QUDT URIs. Artifacts outlined are available and referenced^15^. The final products appropriate for reuse are shown with bold text. QUDT ontology is the units ontology from QUDT v2.1.25.

A multi-step process was used to create a lookup table for mapping raw units and QUDT units (Fig. 1). A corpus of unit descriptions from existing metadata documents was assembled from materials provided by three organizations that manage environmental data. DataONE provides access to data across multiple member repositories to support search and discovery of Earth and environmental data. DataONE provided a tabulation of units and their frequency of use. This tabulation integrates datasets across DataONE member organizations and were drawn from a variety of metadata standards. Full metadata documents using Ecological Metadata Language (EML) were provided by the Environmental Data Initiative (EDI), which is a repository for environmental data, including data from the U.S. Long-Term Ecological Research (LTER) program, and by the National Ecological Observatory Network (NEON), which is a continental-scale observation facility. All metadata were acquired between 2022-10-25 and 2022-11-09^15^.

Raw unit descriptions from the three sources were appended, and the number of uses and the number of organizations tabulated, resulting in a list of distinct units (no duplicates). To group together different representations of the same underlying unit (e.g., gramPerMeterSquared, grams per square meter, g/m2, g m-2), a set of 86 string substitutions were used to create “pseudounits” such as grampermetersquared, to more easily map a unit’s meaning to QUDT. Pseudounits were lowercase, with no spaces or symbols. These were tabulated, and the number of raw units associated with a specific pseudounit calculated to identify units that were used most frequently.

To map raw units to units in the QUDT Units Ontology, a second set of 346 sequential string substitutions was used to transform each pseudounit (e.g., grampermetersquared) into a form that mirrored QUDT units (e.g., GM-PER-M2, see below). The list of substitutions was developed by iteratively adding transformations that addressed the most common pseudounits and their component elements. At each iteration the most common remaining unmatched pseudounits were identified and additional transforms added. The order of substitutions was designed to maximize matching of string fragments within pseudounits, such as when a pseudounit string included strings that were themselves units (e.g., percent is part of gram***percent***imetersquared). Common misspellings were also addressed in the substitution list (e.g., cenimeter, celcius, and cubitmeter).

QUDT’s convention for formatting a unit is abbreviations separated by dashes (“-”)^12^. This convention proved useful in the stepwise conversion because, since the original pseudounits could not contain dashes, it made it easy to distinguish which portions of a pseudounit had already been converted, and which had not. Our sequential string substitutions coupled with some additional processing transformed pseudounits into QUDT units. For example, with the pseudounit grampermetersquaredperday, sequential substitutions were

1. metersquared to “-M2-” to produce gramper-M2-perday
2. day to “-DAY-” to produce “gramper-M2-per-DAY-”
3. gram to “-GM-” to produce “-GM-per-M2-per-DAY-”
4. per to “-PER–” to produce “-GM–PER–M2–PER–DAY-”

Finally, leading and trailing dashes were removed and multiple dashes reduced to single dashes (GM-PER-M2-PER-DAY) and subsequent uses of “-PER-” beyond the first use were converted to dashes to yield GM-PER-M2-DAY, which is a QUDT unit.

Transformations were performed in R (v4.2) and the transformed units were compared with those in QUDT v2.1.25. Pseudounits that were successfully matched to QUDT units were output along with the associated original raw units, the QUDT units, and QUDT URIs and the count of uses in metadata documents^15^. To increase the list of units that could potentially be matched in metadata documents (i.e., beyond those encountered in our original corpus of metadata), additional rows were added to contain all units from QUDT (regardless of whether they were used in the metadata corpus), all UCUM unit descriptions linked to QUDT units that were greater than two characters long (ambiguities were introduced with shorter UCUM codes), and the list of pseudounits (treated as raw units). The table was further enhanced by adding columns of ancillary data drawn from the QUDT ontology, such as labels, the QUDT dimension vector, the multiplier to SI units, unit descriptions and alternative codings (e.g., UCUM). The resulting lookup table allowed any raw unit, pseudounit, QUDT unit or UCUM code that could be mapped to a QUDT unit to be rapidly associated with a QUDT unit (e.g., GM-PER-M2) or Universal Resource Identifier (URI) (e.g., http://qudt.org/vocab/unit/GM-PER-M2). Using the lookup table as their basis, a web service for looking up QUDT units and R functions for adding annotations to EML metadata documents were developed. The web service was implemented using a simple PHP script which ingested the lookup table, compared the raw unit input to entries in the table, and if matched, extracted additional information from the lookup table which was used to produce output in the desired form.

To identify potential errors in the mapping, manual inspections of raw units and their mappings were performed by Information Managers from 24 LTER sites and EDI, who were familiar with the datasets, and in some cases the metadata originators. During the 2023 LTER Information Management Committee meeting in Burlington VT, they were given a list of all the raw units used at their individual sites along with the QUDT mappings, and asked to identify any units that were missed or where the mapping to QUDT was incorrect.

## Results

### Corpus of units

**Fig. 2:**
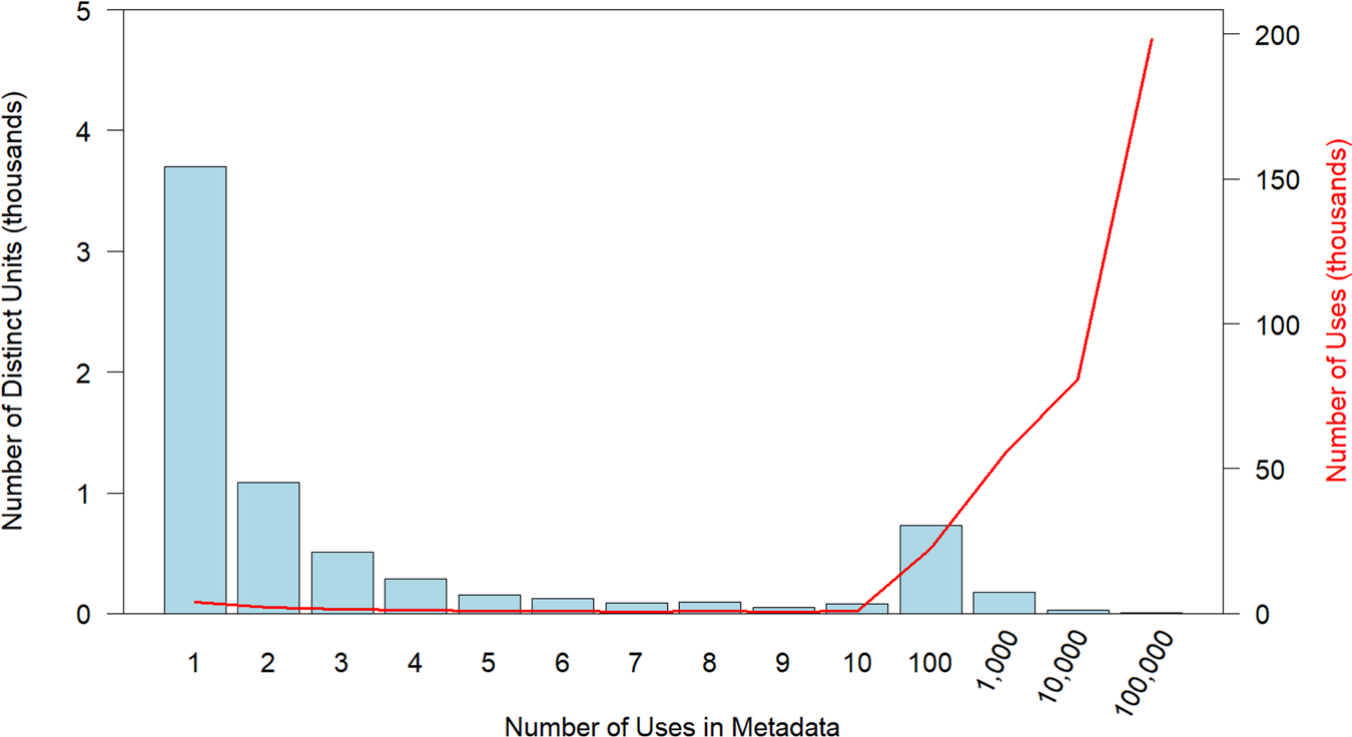
Number of distinct units (bar plot, left axis) and number of uses (line, right axis) versus the number of uses in metadata documents. Units used only once make up the majority of distinct units, but only a small fraction of the number of uses of raw units in metadata documents.

Our corpus of 355,057 raw unit descriptions came from EDI (49.8%), DataOne (45.8%) and NEON (4.4%). Despite the very large number of unit descriptions in the source metadata documents, there were only 7,110 distinct units, and their frequency of use varied widely (Fig. 2). More than 50% of distinct units were used only once, and 74% were used fewer than 3 times (Fig. 2, lower left). Fewer than 10% of distinct units were used more than 15 times, and 3% more than 100 times. Six units (meter, celsius, meters per second, percent, number and dimensionless) accounted for about 17% of uses (more than 10,000 times each, Fig. 2, upper right).

There were some differences in the use of raw units across organizations. Only 49 distinct units were used by all three organizations (DataONE, NEON, EDI), but these were used extensively, accounting for 218,215 (61%) of all unit uses. There were 1,071 raw units used by two of the organizations, which accounted for an additional 111,752 unit uses (31%), leaving 5,990 distinct units used only by a single organization and accounting for only 8% of the raw unit descriptions in metadata. NEON units overlapped almost entirely with the other organizations, with only 15 (<1%) distinct units not used by others. In contrast, DataONE had 4,702 distinct units (81%) that were not used by either of the other two organizations, and EDI featured 1,273 (53%) distinct units used only within EDI metadata.

We did not attempt a quantitative analysis of the raw units used only once by a single organization. However, cursory examination indicated that a large number of infrequently used putative units were not units at all, but instead were numeric, labels, location names, or other terms relevant to data collection, but misplaced within the metadata. There is seemingly little benefit in trying to link non-units to a unit ontology so we focused our efforts instead on those units that were widely applied.

### Pseudounits & QUDT units

Following the procedures diagrammed in Fig. 1, pseudounits had been derived from distinct units using 107 string substitutions, which resulted in a small reduction (14%) in the total number of distinct units as pseudounits (Table 1). After an additional 353 stepwise string transformations of pseudounits into QUDT units, fewer than 10% of the pseudounits (12.6% of distinct units) were matched to QUDT units, (Table 1). However, when matched to the uses of raw units in metadata, over 90% of unit uses were matched to QUDT units. Among the organizations, we were able to map 96%, 93%, and 89% of NEON, EDI, and DataONE raw units, respectively. There were several hundred distinct units (including 72 used more than 10 times in raw units) which did not match a unit in QUDT. These will be reviewed, and result in a list of candidate units for QUDT.

**Table 1:** Summary of units.

Type of Units	Count (Percent)
Raw units (duplicates allowed)	355,057
Distinct units (duplicates removed)	7,110
Distinct units matched to QUDT	896 (12.6%)
Raw units matched to QUDT	324,811 (91.4%)

### Manual checking

In the review of terms by 24 data managers, only one potential error was identified, related to a QUDT unit name that did not follow the established pattern; MicroG refers to a unit of acceleration (microgravity) but MicroG-PER-CentiM2 refers to mass per unit area, although MicroGM-PER-CentiM2 would be the internally consistent representation. Additionally, they identified 99 additional potential matches and 136 cases where units would need to be added to QUDT. We are collaborating with QUDT to make those additions. Unit additions that successfully resolve to QUDT units will ultimately be added to the lookup table.

### Incorporating the QUDT ontology into dataset metadata

The mapping of raw units to units in an ontology is most useful when the mapping is a resource for researchers or data managers. A simple method of going from a raw unit or unit in another coding scheme (e.g., UCUM) to a unit in the QUDT ontology is needed. To this end, a lookup table containing columns: unit to be matched, the equivalent QUDT Unit, and additional material (e.g., Label, URI, DimensionVector) drawn from QUDT were created. Anticipating future use, the table was expanded with rows for (1) all 896 of the successfully mapped, original raw units, (2) all the units in QUDT (1,632), (3) the list of successfully-mapped pseudounits, and (4) all the UCUM codes listed in QUDT that were greater than two characters in length (1,571).

**Fig. 3:**
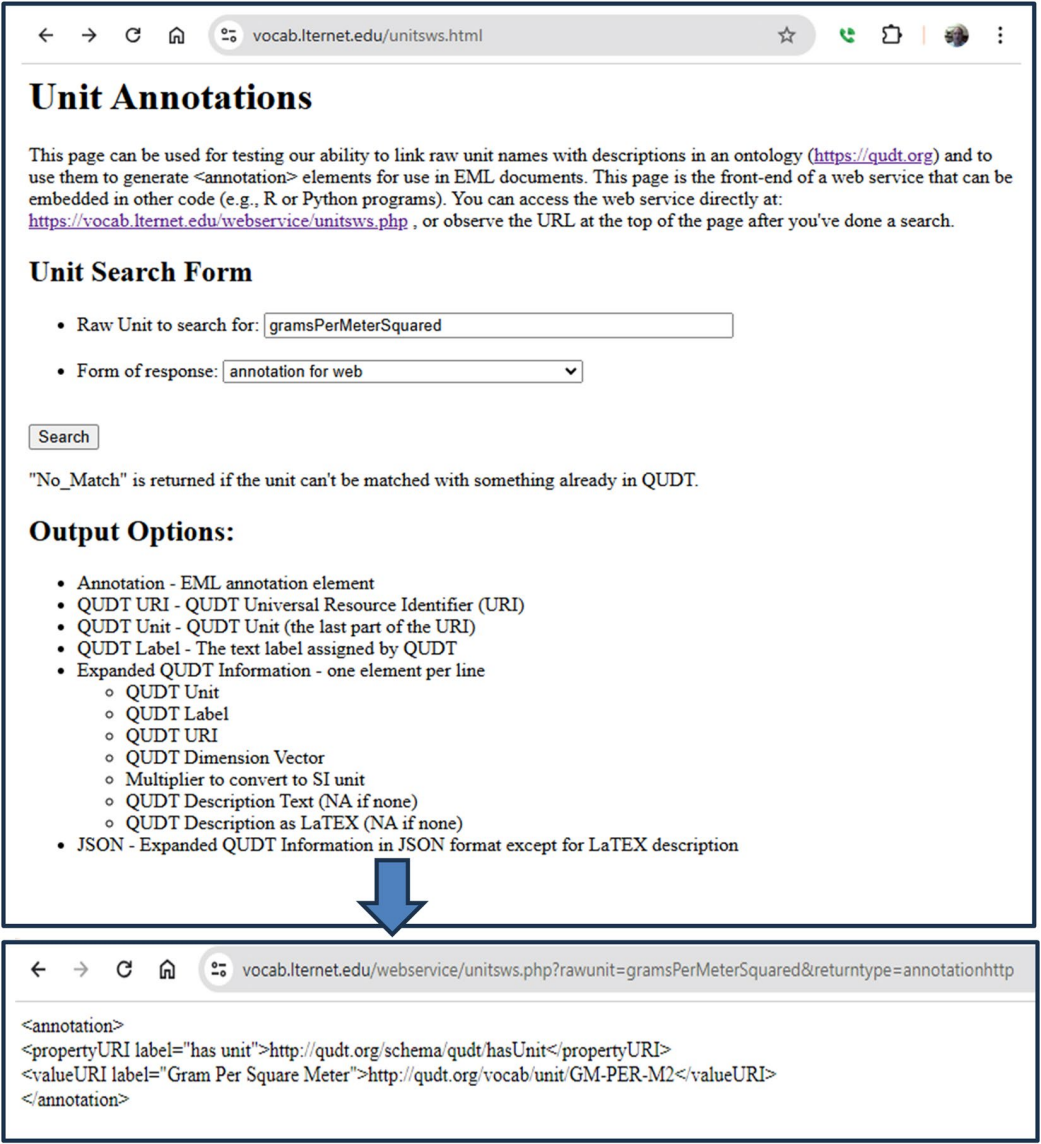
Web page for testing the web service. The web service can be exercised without using the web page by including the parameters rawunit and returntype directly. The resulting <annotation> element (bottom) which can be added to an EML 2.2 metadata document.

Two tools were created that draw upon the lookup tables to facilitate adding QUDT units to EML metadata as annotations^16^. The first is a REST-based web service (https://vocab.lternet.edu/webservice/unitsws.php, accessed 2024-12-18) in which a user-supplied unit is queried in the lookup table and selected QUDT content (QUDT ID URI, label, etc), output in various forms (html, xml, json). Calls to this web service can be embedded into user code or accessed directly using a web-page front-end. (Fig. 3, https://vocab.lternet.edu/unitsws.html, accessed 2024-12-18).

**Fig. 4:**
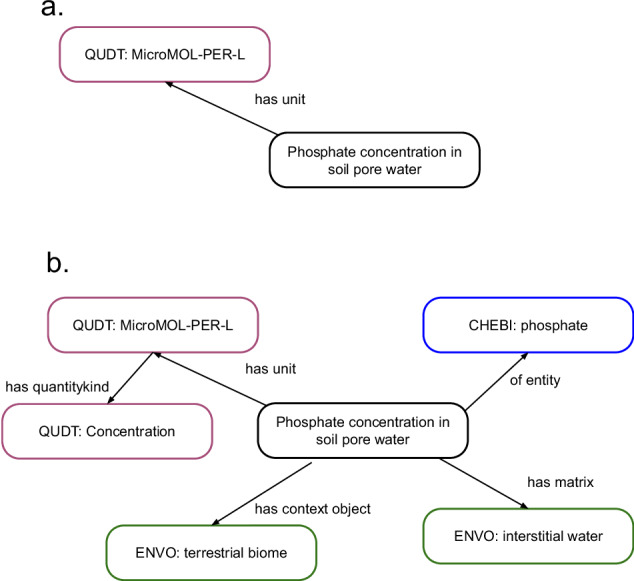
Conceptual RDF “triples” that could be added to metadata for a measurement of “phosphate concentration in soil pore water” as an annotation. (**a**) Triple specifying that it uses the unit “MicroMOL-PER-L” from the QUDT ontology. (**b**) three additional triples showing what was measured (phosphate), its matrix and context (terrestrial biome and interstitial water, respectively). The QUDT ontology provides for an additional inferred annotation: through its unit, the measurement is associated with the quantity kind “concentration”. Terms derived from different ontologies are shown in different colors.

A second tool that draws on the lookup table is a set of R functions that reads existing EML metadata to generate an edited copy of the document with annotation elements added. Optionally, it can update other metadata as required by the host repository, to increment the package version and add “id” attributes to EML <attribute> tags if they are not already provided. Annotation elements in EML provide a way to provide Resource Description Framework (RDF) triplets relating a specific measured environmental attribute to a unit in the QUDT ontology (Fig. 4a). Doing so allows queries to retrieve information on attributes that share common units, for a wide variety of *ad hoc* representations of units provided in the <unit> element of EML.

## Discussion

For metadata schemas that permit annotation, using annotations to link human-readable units to an ontology is a powerful way to help facilitate automation of ecological data processing. It allows flexibility to accommodate human preferences for particular representations of units while allowing a strict and consistent machine-interpretable representation. Moreover, an ontology connects unit concepts with other measurement concepts, such as kinds of quantities and dimensions, which increases their findability and interoperability. These interconnections may serve as a springboard to improve automation of data integration and processing. Steinberg *et al*.^17^ characterized 16 use cases (UC) for the use of unit ontologies and presented criteria for assessing ontologies relative to their suitability for those use cases. Here we anticipate that the addition of new unit annotations to metadata will enhance the utility of datasets through (1) improvements in automated conversion of units (UC 5 in^17^), (2) greater discoverability by enabling search for datasets that share units or dimensions (UC 15), (3) enhanced interpretability through access to labels and descriptions, enabling dimensional analysis of equations (UC 7), and (4) identifying equivalency in units across systems (UC 12). For example, Hippolyt *et al*.^18^ examined how standardized measurement data and metadata could be presented using semantic web technologies for use cases in Earth observation and bathymetry. Despite some limitations, they were able to demonstrate machine-actionability by retrieving information from the dataset descriptions.

Despite the generally successful mapping of existing units in the three-organization corpus to QUDT, we encountered some gaps in the QUDT coverage. QUDT has a well-defined methodology for proposing additional units, and we are developing submissions to help eliminate the gaps we identified. We estimate that the proposed additions to QUDT will add an additional 1-2% of coverage. Roughly 7% of units may never be matched to QUDT. Many of the unmatched units are nonsensical such as “4a” or “atlantis/alvin” or “martha stewart” or “−9999.” In many cases, we believe these terms actually belong in other parts of the metadata (e.g., methods, missing values, lists of codes and locations).

We were encouraged by the efficacy of using sequential search-and-replace operations to convert pre-existing, raw units into QUDT-compatible unit codes, especially given the diversity of raw units. The highly skewed distribution of uses of individual units shown in Fig. 2, where a small subset of raw units was used very frequently in metadata, but most raw units were seldom used led to a situation where a low percentage of distinct units matched, but a high percentage of raw units matched. Part of what made this possible was that many of the frequently used raw units followed a convention of some sort. Some unit descriptions used a list of predefined units (part of the EML schema), or written unit names styled per EML conventions. Common conventions included widely recognized abbreviations (e.g., m/s) or exponents (ms-1). The challenge was that the corpus of metadata included many different conventions. Given this skewed distribution of unit descriptions, tallying the number of uses of particular unit formulations, as simplified lowercase pseudounits, may not have been necessary, because we may have been able to map directly to raw units using their conventions. However, doing so helped us focus on units that were used most frequently, enabling us to link 91% of the units to QUDT.

A particular difficulty is the conflation of units with other aspects of measurements, for example, an observation that combines nouns with a unit. For example, “milligrams of nitrogen” may be the name of a measurement or in the QUDT model, a Quantity^12^ but the unit is simply milligrams. There are advantages to separating units and measurements (quantities). For the work presented here, addressing only the units allowed us to scope the project realistically. A list of measurements would have been enormously long because of the number of potential combinations of subjects and units. In the context of QUDT, the dimension vector controls what unit conversions can be performed, i.e., direct conversions are possible only between units with matching dimension vectors. This ensures that conversions such as between milligrams per liter and millimoles per liter cannot be performed with the unit alone, since one must know the chemical substance and its atomic weight – measurement features that are part of the quantity rather than the unit.

QUDT provides “concept qualifiers,” which are separated from units by an underscore. These are used primarily to clarify the source of units and to distinguish between similar units in different systems (e.g., GAL_IMP, GAL_UK, GAL_US distinguishing whether measurements are in imperial, United Kingdom, or United States gallons). But it also contains a small number of units where the concept qualifier is a substance such as GM_Carbon-PER-M2-DAY or CentiM_H2O that really represent measurements. These are generally for legacy units from QUDT’s origin, and this practice is not currently recommended because such a context-specific specialization edges toward the realm of a qudt:Quantity rather than a dedicated unit (S. Ray pers. comm.). UCUM provides a similar convention where additional information can be provided in braces as part of the unit specification such as mm{H2O} or mm{water} for millimeters of water. However, terms in braces are considered annotations designed for human-readability and are not defined within UCUM.

Use of domain-specific measurement frameworks would provide definite advantages in many contexts, such as for conversions that require additional knowledge (e.g., as described above for molecular weight). Similarly, a measurement such as “number of stems per square meter of forest” is not readily comparable to “number of fish per square meter of ocean;” even though the unit “number per square meter” is the same, their subjects are unrelated. In these cases, the complete context of the measurement is essential, but would have been only partly accommodated by the QUDT (and UCUM) practice of adding qualifiers.

There are many efforts to create measurement ontologies; O&M^19^, OBOE^20^ and I-Adopt^21^ all supply the framework to hold needed context for scientific measurement (as well as units). However their creation can be complex and current use is insufficient to adequately test the models. In our user communities of data managers, we are considering the use of multiple annotations in EML metadata to supply needed context. These could take the form of additional annotations (as in Fig. 4b) that contain relationships to measurement concepts like subject (e.g., “nitrogen”, “plant stems”, “fish”) or context (“forest”, “ocean”). The result would be a collection of RDF triplets associated with a measured attribute that included a unit specification as well as the important entities. For example, annotations for “phosphate” and “interstitial water” could be associated with MilliMOL-PER-L to get “phosphate concentration in pore water” (Fig. 4b). Ontologies already exist for many of those concepts and are designed to be incorporated into other frameworks^22–24^.

In addition to allowing a more gradual adoption of measurement annotation with ontological concepts, we expect that this practice would provide a corpus of annotated table columns that could be used to build dictionaries of measurements. A sizable number of measurement components (e.g., units, chemicals, things being measured, context milieu, phenotypic traits) appearing in dataset annotations would facilitate construction of true dictionaries of important, commonly used measurements. There are multiple benefits to such a strategy: it would help to drive ontology adaptation and maintenance, help test the usability of observational models such as O&M, OBOE and I-ADOPT, and encourage development of extensions to serve other measurement attributes such as domain, range and observational or analysis methods. Further, it would illustrate the value of ontology for other aspects of dataset use (e.g., search and discovery). Making units machine-readable is an important first step and has value in itself, but it will become even more valuable when combined with additional annotations.

Figure 4b also illustrates an additional inferred annotation: through its unit, this measurement is associated explicitly with the quantity kind “concentration”. Faceted search by quantity is likely to be useful for querying a diverse data corpus - to yield, for example, datasets containing measurements of “primary production”, even if that term does not appear explicitly in dataset metadata - and QUDT Quantity Kind could be used for this. Although QUDT’s unit vocabulary has reasonable coverage for environmental science, its vocabulary of Quantity Kinds is noticeably focused on the domains of engineering and aeronautics, and is incomplete for biology and environmental science. Our initial submission to QUDT will focus on defining new units and a minimum number of Quantity Kind instances. But as query requirements become more clearly defined, we anticipate a need to create additional instances for environmental quantities with relationships added to existing units.

In conclusion, linking of raw, *ad hoc* units in a large corpus of existing ecological metadata to the QUDT units ontology using an ordered set of string transformations proved to be more efficient than anticipated initially. Although a large number of infrequently used, raw units were not matched, we were able to match ~91% of unit instances identified among metadata collected from DataONE, the EDI, and NEON to the QUDT ontology. A lookup table containing alternative representations of units from a variety of sources (raw units, QUDT units, UCUM codes) made it possible to develop web services and R code to annotate existing EML metadata. Use of such a table is not limited to EML and might be applied to annotating metadata using other standards as well. Providing such annotations, linked to an ontology, provides additional opportunities for improving the usability of environmental data.

## Data Availability

Raw and derived data are available as open data via the Environmental Data Initiative with the identifier 10.6073/pasta/03de5f726541999caeed8ef2a55e8c3c.

## Code availability

Copies of the R (v. 4.2) code used in the analysis and processing are available in ent9_UnitAnalysisRCode.zip contained in the dataset: Porter, J., M. O’Brien, M. Frants, S. Earl, M. Martin, and C. Laney. 2024. Mapping EDI, NEON and DataONE units to the QUDT ontology, 2022 ver 2. Environmental Data Initiative. 10.6073/pasta/03de5f726541999caeed8ef2a55e8c3c (Accessed 2024-08-28) and at https://github.com/EDIorg/Units-WG/tree/main/RCode_JP.
